# Trajectory-Based Identification of Rotary-Axis Position-Independent Geometric Errors Considering Excitation Projection Effects

**DOI:** 10.3390/mi17020256

**Published:** 2026-02-16

**Authors:** Songtao He, Seth Osei, Wei Wang, Jiaying Wang, Kaiyuan You, Qicheng Ding, Jiahao Yu

**Affiliations:** School of Mechanical and Electrical Engineering, University of Electronic Science and Technology of China, Chengdu 611731, China

**Keywords:** PIGEs, rotary axis, five-axis machine tool, trajectory, identification accuracy

## Abstract

Improving the identification accuracy of geometric errors in five-axis machine tools is a critical requirement in advanced manufacturing. Although rotary axes enhance machining flexibility and productivity, they introduce additional geometric errors, among which position-independent geometric errors (PIGEs) are a dominant source of accuracy degradation. Existing studies have paid limited attention to how excitation projection associated with test trajectories affects identification accuracy. This study proposes a trajectory-based identification method using a single setup and systematically investigates the influence of excitation projection on the identification accuracy of rotary-axis PIGEs. An error model and a double differential identification scheme are developed and validated through simulation and experimental studies. For the AC-type machine tool, both simulation and experimental results demonstrate that the accurate identification of all rotary-axis PIGEs is achieved after the second differential under a favorable excitation projection. In contrast, the simulation results for a BC-type machine tool indicate that the optimal excitation projection differs due to its kinematic configuration. Compensation results further confirm the effectiveness of the identified PIGEs, showing a significant reduction in trajectory errors. The results reveal that identification accuracy is governed by the relationship between excitation projection and machine tool structural configuration rather than by the physical test trajectory itself. The proposed method, which requires only a single setup, provides an effective and practical approach for improving the identification accuracy of rotary-axis PIGEs in five-axis machine tools.

## 1. Introduction

High-speed and ultra-precision machining demands high geometric accuracy from machine tools to ensure surface quality and dimensional integrity. While five-axis machine tools provide the flexibility required for machining complex parts, the addition of rotary axes increases the number and complexity of geometric errors, which contribute to both quasi-static and dynamic machine tool errors [1]. Geometric errors constitute a major portion of the total machining error, contributing up to 40–50% overall and as much as 78.7% in some studies. These errors are commonly classified into position-dependent geometric errors (PDGEs), arising from motion and component defects, and position-independent geometric errors (PIGEs), primarily caused by assembly and linkage imperfections. Several studies have demonstrated that compensating PIGEs alone can recover up to 70–79% of machining accuracy [2,3,4,5,6], indicating that PIGEs, particularly those associated with the rotary axes, are among the most critical error sources affecting five-axis machine tool accuracy.

Various measurement devices and strategies have been employed to identify PIGEs in rotary axes, including double ballbar (DBB), touch-trigger probes, R-test devices, artifacts, laser trackers, CapBall systems, and laser interferometers [5,7,8,9,10,11,12,13,14,15,16,17]. Among these, the R-test device has demonstrated high precision, robustness, and efficiency, particularly under dynamic measurement conditions, making it well-suited for rotary-axis error identification [10,12]. Because some geometric errors cannot be directly measured, model-based identification approaches are widely used. Homogeneous transformation matrices (HTMs), combined with numerical identification schemes such as least squares, Chebyshev polynomials, Gauss–Newton methods, and other kinematic modeling techniques, have been extensively applied due to their effectiveness in representing rigid-body motion in three-dimensional space [18,19,20,21,22]. Direct measurement approaches, which isolate and measure individual axis errors, are accurate but often time-consuming, costly, and impractical for full machine calibration [6,16,23,24]. Consequently, indirect identification methods that simultaneously excite multiple axes have been widely adopted to reduce measurement complexity [7,25,26]. Most PIGE identification methods typically rely on multiple setup points, auxiliary fixtures, or multiple test trajectories to achieve full parameter identification. Methods based on DBB, CapBall, or artifact measurements often require extension bars or explicit rotational-axis identification, which increases setup complexity and sensitivity to thermal and alignment errors [27,28,29,30,31,32,33]. These limitations motivate the development of identification methods that minimize setup complexity while maintaining high identification accuracy.

Trajectory-based measurement approaches have emerged as an attractive alternative, as they allow single-setup identification by moving the tool along predefined paths such as ISO AK-, BK-, CK-series trajectories and 8-shaped RTCP paths; however, many still depend on multiple trajectories or exhibit limited parameter observability, leading to reduced identification accuracy for certain PIGEs [34,35,36]. Several studies have employed differential or trajectory-based strategies to reduce setup requirements [12,37,38,39]. However, existing trajectory-based methods often suffer from limited identification accuracy, restricted axis motion ranges, or the need for multiple trajectories to identify all rotary-axis PIGEs [15,40,41,42]. Moreover, most previous studies implicitly assume that the physical test trajectory alone determines the identification sensitivity, while the influence of how trajectory-induced errors are projected into the measurement frame has received limited attention. In practice, identification accuracy is governed not only by the test trajectory itself but also by the observability of the error parameters, which depends on the relationship between excitation characteristics and the machine-tool kinematic configuration. In contrast, the method proposed in this study aims to improve identification accuracy under a single setup by explicitly considering the relationship between trajectory-induced excitation and machine-tool kinematic configuration, thereby enhancing the observability of all rotary-axis PIGEs without introducing additional hardware or measurement steps.

To address this gap, this study proposes a trajectory-based identification method using a double differential scheme to identify all eight PIGEs in the two rotary axes of a five-axis machine tool under a single setup. Rather than modifying the physical test trajectory, the proposed approach investigates how excitation projection influences identification accuracy in relation to machine tool configuration. The method is validated through simulation and experimental studies on AC-type machine tools, with additional simulations conducted on BC-type configurations to generalize the findings. The results reveal that identification accuracy is strongly governed by excitation observability conditioned by machine topology, providing new insight into trajectory-based geometric error identification and offering practical guidelines for improving rotary-axis PIGE calibration in five-axis machine tools. The rest of this article is arranged as follows: the machine tool structure and error modeling are presented in Section 2. The simulation and experimental processes are presented in Section 3. The results are discussed in Section 4, and the work is concluded in Section 5.

## 2. Machine Tool Structure and Error Modeling

### 2.1. Machine Tool Structure

A five-axis machine tool with a tilting table (machine code: w C’Aʹ b X Y Z (C1)t) is considered in this work for both simulation and experimental processes; the machine code is defined by ISO 10791-6 [35] where the cutting tool, the workpiece, and the machine bed are represented “t”, “w”, and “b” consisting of two chains: workpiece and cutting-tool chains.

**Figure 1:**
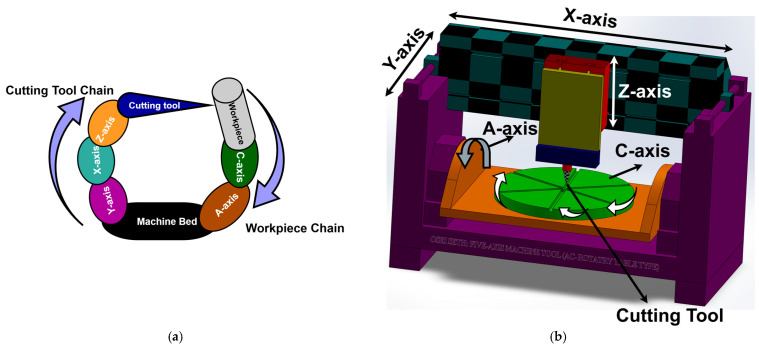
A five-axis machine tool with tilting table: (**a**) topology; (**b**) structural configuration.

**Figure 2:**
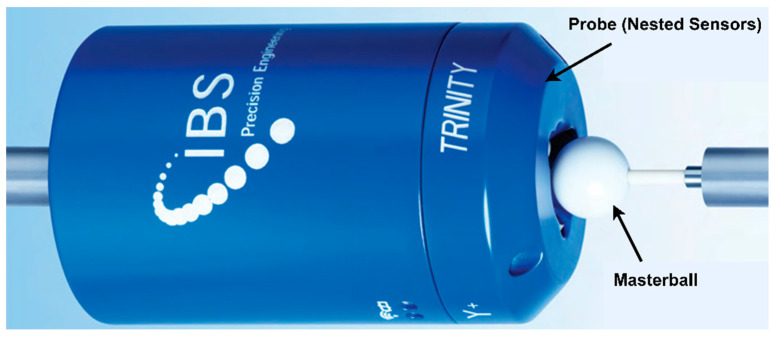
IBS precision engineering rotary inspector (R-test device).

The topology of the machine tool and its structural configuration are shown in Figure 1a,b, respectively. The IBS Rotary Inspector device [40] is used as the measuring instrument in this work due to its high precision ability and 3D error dimensionality, as shown in Figure 2.

It has a precision sphere and three nested sensors, which help give the error in X, Y, and Z directions, and the device specifications are presented in Table 1.

### 2.2. Modeling of PIGEs

Since the PIGEs in two rotary axes are the focus of this study, the modeling process considers only the workpiece chain of the machine tool presented in Section 2.1. The mathematical model of the five-axis machine tool is developed using the homogeneous transformation matrix theory by constructing the relative motion matrix between the axes.

(1) Tji=Ti+1i  .i+2i+1T.i+3i+2T…Tj  j−1

i≥0 and j≥1,2,…,i

where the transformation from frame i to j is represented as Tji. Considering only the rotary axes, the transformation matrices of the linear axes are set to unity, and they will not have any mathematical influence on the model; hence, they are ignored during error modeling. The definition of parameter representation of the PIGEs is defined in Table 2. Setting the absolute coordinate on the machine tool bed, the transformation from the absolute coordinate (r) to the workpiece coordinate (w) can be expressed as:

(2) Twra=ξATA.ξCTC.TwC

(3) Twri=TA.TC.TwC

(4) TwC=[wx wy wz 1]T

$$T_{A}=\left[\begin{array}{cccc} 1 & 0 & 0 & 0\\ 0 & cos(\theta_{a}) & -sin(\theta_{a}) & 0\\ 0 & sin(\theta_{a}) & cos(\theta_{a}) & 0\\ 0 & 0 & 0 & 1 \end{array}\right],\;\xi_{A}=\left[\begin{array}{cccc} 1 & -\beta_{za} & \beta_{ya} & 0\\ \beta_{za} & 1 & 0 & \delta_{ya}\\ -\beta_{ya} & 0 & 1 & \delta_{za}\\ 0 & 0 & 0 & 1 \end{array}\right]$$

$$T_{C}=\left[\begin{array}{cccc} cos(\theta_{c}) & -sin(\theta_{c}) & 0 & 0\\ sin(\theta_{c}) & cos(\theta_{c}) & 0 & 0\\ 0 & 0 & 1 & 0\\ 0 & 0 & 0 & 1 \end{array}\right],\;\xi_{C}=\left[\begin{array}{cccc} 1 & 0 & \gamma_{yc} & \delta_{xc}\\ 0 & 1 & -\gamma_{xc} & \delta_{yc}\\ -\gamma_{yc} & \gamma_{xc} & 1 & 0\\ 0 & 0 & 0 & 1 \end{array}\right]$$

where the actual (with PIGEs) and ideal (without PIGEs) transformations are defined as Twra and Twri respectively, TwC represents the transformation from the C axis to the workpiece, $\theta_{a}$ and $\theta_{c}$ are the respective angular positions of the A and C axes. The error between the actual and ideal trajectory can then be expressed as:

(5) Eerr=Twra−Twri

(6) $$E_{err}=\left[\begin{array}{c} \delta_{xc}+\gamma_{yc}\;w_{z}+\beta_{ya}\;\delta_{yc}\;\sin\left(\theta_{A}\right)+\beta_{ya}\;w_{z}\;\cos\left(\theta_{A}\right)+\beta_{za}\;w_{z}\;\sin\left(\theta_{A}\right)-\beta_{za}\;\delta_{yc}\;\cos\left(\theta_{A}\right)+\beta_{ya}\;w_{x}\;\sin\left(\theta_{A}\right)\;\sin\left(\theta_{C}\right)\\ +\beta_{za}\;\gamma_{xc}\;w_{z}\;\;\cos\left(\theta_{A}\right)-\beta_{ya}\;\gamma_{xc}\;w_{z}\;\sin\left(\theta_{A}\right)-\beta_{za}\;w_{y}\;\cos\left(\theta_{A}\right)\;\cos\left(\theta_{C}\right)-\beta_{za}\;w_{x}\;\cos\left(\theta_{A}\right)\;\sin\left(\theta_{C}\right)+\beta_{ya}\;w_{y}\;\cos\left(\theta_{C}\right)\;\sin\left(\theta_{A}\right)\\ -\beta_{ya}\;\gamma_{yc}\;w_{x}\;\cos\left(\theta_{A}\right)\;\cos\left(\theta_{C}\right)+\beta_{ya}\;\gamma_{xc}\;w_{y}\;\cos\left(\theta_{A}\right)\;\cos\left(\theta_{C}\right)+\beta_{ya}\;\gamma_{xc}\;w_{x}\;\cos\left(\theta_{A}\right)\;\sin\left(\theta_{C}\right)-\beta_{za}\;\gamma_{yc}\;w_{x}\;\cos\left(\theta_{C}\right)\;\sin\left(\theta_{A}\right)\\ +\beta_{ya}\;\gamma_{yc}\;w_{y}\;cos(\theta_{A})\;sin(\theta_{C})+\beta_{za}\;\gamma_{xc}\;w_{y}\;cos(\theta_{C})\;sin(\theta_{A})+\beta_{za}\;\gamma_{xc}\;w_{x}\;sin(\theta_{A})\;sin(\theta_{C})+\beta_{za}\;\gamma_{yc}\;w_{y}\;sin(\theta_{A})\;sin(\theta_{C})\\ \delta_{ya}+\beta_{za}\;\delta_{xc}+\delta_{yc}\;\cos\left(\theta_{A}\right)+\beta_{za}\;w_{x}\;\cos\left(\theta_{C}\right)-\gamma_{xc}\;w_{z}\;\cos\left(\theta_{A}\right)-\beta_{za}\;w_{y}\;\sin\left(\theta_{C}\right)+\beta_{za}\;\gamma_{yc}\;w_{z}\\ -\gamma_{xc}\;w_{x}\;sin(\theta_{A})\;sin(\theta_{C})-\gamma_{yc}\;w_{y}\;sin(\theta_{A})\;sin(\theta_{C})+\gamma_{yc}\;w_{x}\;cos(\theta_{C})\;sin(\theta_{A})-\gamma_{xc}\;w_{y}\;cos(\theta_{C})\;sin(\theta_{A})\\ \delta_{za}-\beta_{ya}\;\delta_{xc}+\delta_{yc}\;\sin\left(\theta_{A}\right)-\beta_{ya}\;w_{x}\;\cos\left(\theta_{C}\right)+\beta_{ya}\;w_{y}\;\sin\left(\theta_{C}\right)-\gamma_{xc}\;w_{z}\;\sin\left(\theta_{A}\right)-\beta_{ya}\;\gamma_{yc}\;w_{z}\\ -\gamma_{yc}\;w_{x}\;cos(\theta_{A})\;cos(\theta_{C})+\gamma_{xc}\;w_{y}\;cos(\theta_{A})\;cos(\theta_{C})+\gamma_{xc}\;w_{x}\;cos(\theta_{A})\;sin(\theta_{C})+\gamma_{yc}\;w_{y}\;cos(\theta_{A})\;sin(\theta_{C}) \end{array}\right]$$

## 3. Results

From the model constructed in the previous section, the PIGEs can be computed by taking the first-order partial derivates of the error with respect to the eight (8) PIGEs as differential variables:

(7) $$E_{err,j}={\omega_{j}.\varphi }_{j}$$

j=1,2,3,…,n

$$\omega_{j}={\left[\frac{\partial E_{err,X,j}}{\partial \varphi },\frac{\partial E_{err,Y,j}}{\partial \varphi },\frac{\partial E_{err,Z,j}}{\partial \varphi }\right]}^{T}$$

$$\varphi_{j}={\left[\begin{array}{cccccccc} \beta_{ya} & \beta_{za} & \gamma_{xc} & \gamma_{yc} & \delta_{ya} & \delta_{za} & \delta_{xc} & \delta_{yc} \end{array}\right]}^{T}$$

where the Jacobian matrix relating the PIGEs and the error, $E_{err}={\left[E_{err,1},E_{err,2},\ldots ,E_{err,n}\right]}^{T}$, for n number of points is represented as $\omega_{j}$ at point j. The PIGEs are computed using the least squares method at $10^{-6}$ tolerance by solving for φ as:

(8) $$\varphi ={{(\omega }^{T}\omega )}^{-1}\omega^{T}E_{err}$$

Though the least-squares method is a good approximator, it can provide inaccurate results based on different factors like the non-linearity of the variables [42]. Henceforth, the PIGEs that are accurately predicted are substituted back into the model, and Equation (7) is differentiated with respect to the inaccurate PIGEs until all eight PIGEs are accurately predicted. For example, if $\beta_{ya}$, $\beta_{za},$ and $\gamma_{xc}$ are accurately predicted in Equation (8) (first differential), they are substituted into Equation (7) to predict the rest, and the new differential variable (second differential) in Equation (8) will be $\varphi_{j}=[\gamma_{yc}\;\delta_{ya}$ $\delta_{za}$ $\delta_{xc}$ $\delta_{yc}]$. The updated PIGEs become $\beta_{ya}$, $\beta_{za},\;\gamma_{xc}$, and all the PIGEs are accurately predicted after the second differential, and/or this is repeated until all PIGEs are accurately predicted.

The root mean square error (RMSE) is used as a comparative tool to evaluate the identification accuracy between two data points $\left(t_{j}\;and\;s_{j}\right)$ in this work. Data points $t_{j}(t_{x},t_{y},t_{z})$ are expressed in Euclidean norm discussed in the reference [43] during evaluation works as:

(9) $$t_{j}=\sqrt{{t_{x}}^{2}+{t_{y}}^{2}+{t_{z}}^{2}}$$

The root mean square is expressed as:

(10) $$RMSE=\sqrt{\frac{\sum_{j=1}^{n}{\left(t_{j}-s_{j}\right)}^{2}}{n}}$$

j=1,2,3,…,n

**Figure 3:**
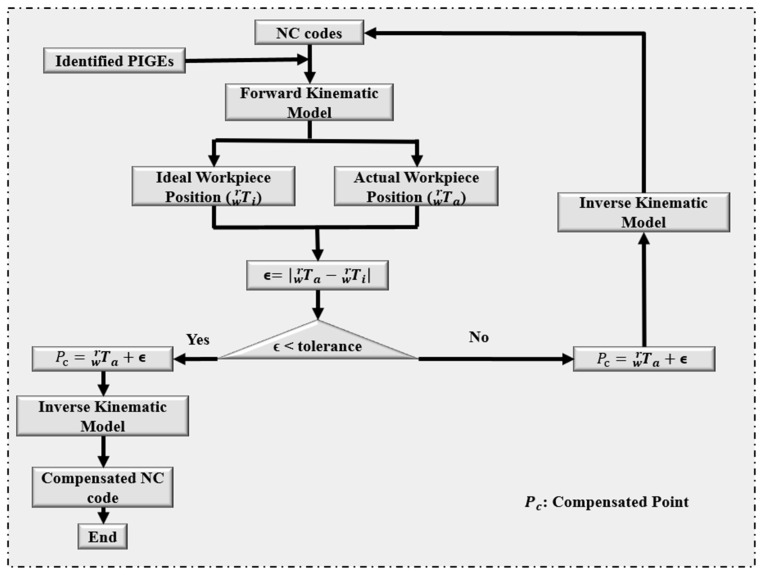
Flowchart of compensation scheme.

The ISO BK3 and the 8-shaped trajectories are employed in this work as test paths; moreover, the impact of the orientations of these trajectories on the identification accuracies is further investigated. Angles 10° and 30° were, respectively, chosen as the angles between the base circle and the table surface, and the cone apex angle as recommended by ISO 10791-6 [35]. The NC code is modified to compensate for the identified PIGEs to validate the effectiveness and accuracy of the method proposed in this study. The compensation scheme of Zha et al. [44] is applied in this work, and the flowchart is presented in Figure 3.

The proposed strategy is implemented through experimental and simulation works, and some assumptions and factors considered are summarized below:

(1) The PDGEs (position-dependent geometric errors) are pre-compensated based on the results obtained in the authors’ previous work [45] using the compensation strategy discussed above. Henceforth, the PDGEs are not considered again during modeling or represented as unit matrices, and only the PIGEs of the rotary axes are considered in this work.
(2) The rotational tool center point (RTCP) test is employed here to compensate for the tool length during experimentation by turning on the RTCP function of the machine tool.
(3) All experimental works were conducted under no-load and quasi-static conditions specified by the ISO [46].
(4) To eliminate or minimize operational errors, environmental influence, and other error sources, all experiments are conducted under a thermally controlled environment (20 °C).
(5) To ensure thermal stability, the machine tool is warmed up for 20 min before conducting experiments.
(6) Thirty (30) measuring points were chosen for each trajectory in both the simulation and experimental processes.

The simulation study is intended to evaluate the identifiability and sensitivity of PIGEs under controlled conditions by introducing known PIGEs into the kinematic model. It is not designed to model time-varying motion errors such as thermal drift or wear-induced effects, which are difficult to parameterize and lie outside the scope of PIGE identification. These practical influences are instead addressed through experimental validation to verify the robustness and effectiveness of the proposed method under real machining conditions.

### 3.1. Simulated Work

A kinematic model of the five-axis machine tool with the same structure described in Section 2 was developed in MATLAB 2024a and used for all simulation studies. The proposed identification method was implemented within this environment, and its effectiveness was evaluated by comparing the preset position-independent geometric errors (PIGEs) with the identified results.

In the simulations, the PIGEs were generated using a Fourier-series-based formulation due to its periodicity and orthogonality properties, expressed as:

(11) $$F\left(k\right)=m_{0}+\sum_{i=1}^{j}\left[m_{i}\sin(k\omega i)+n_{i}\sin(k\omega i)\right]$$

where k∈[1,5] for orientational errors and k∈[1,4] for offset errors, and $m_{0},m_{i},m_{i}\in \left[-0.002,0.002\right]$ are randomly assigned coefficients. The preset PIGEs were selected to reflect realistic industrial conditions, with orientational errors of ±1–15 μrad and translational offsets of ±0–1.0 μm, consistent with reported rotary-axis assembly errors [27,28,29,30,31,32,33]. The ISO BK3 trajectory defined in ISO 10791-6 [35], and the 8-shaped trajectory based on the RTCP test [47] were employed as nominal test paths. Trajectory points were converted into NC commands through forward kinematic transformation.

**Figure 4:**
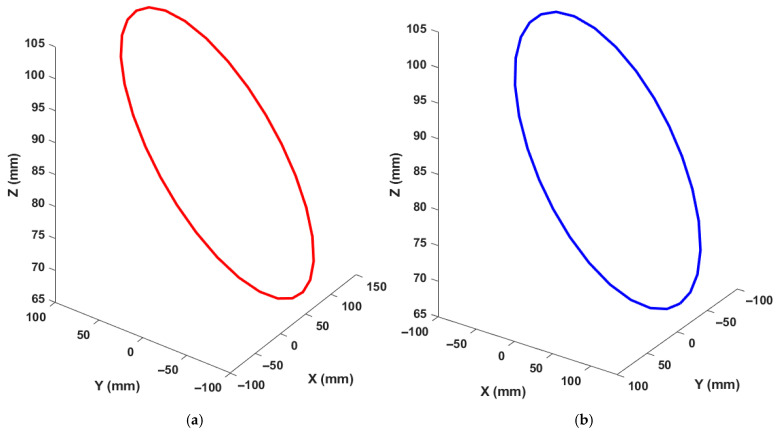
ISO BK3 trajectory: (**a**) tilted around the *X* axis; and (**b**) tilted around the *Y* axis.

**Figure 5:**
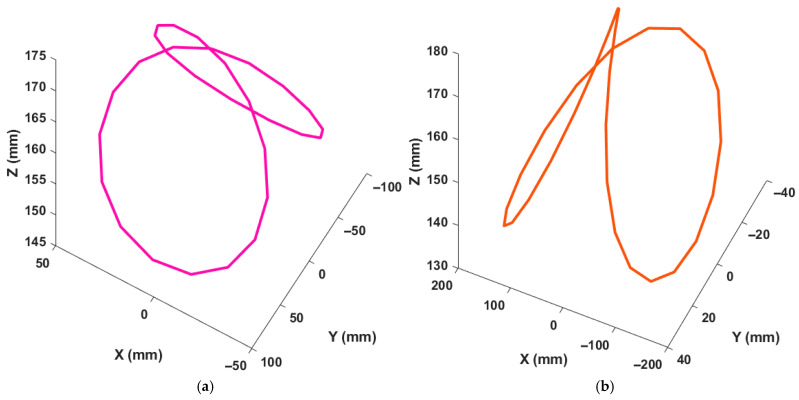
The 8-shape trajectory positions: (**a**) in the X direction; (**b**) in the Y direction.

To investigate the influence of excitation projection on identification accuracy, two measurement-frame excitation configurations were considered for each trajectory while maintaining the same physical tool motion. Specifically, the nominal trajectory was analyzed in its original configuration (Figure 4a and Figure 5a), and an alternative excitation projection was obtained by transforming the measurement frame using homogeneous transformation matrices, resulting in a different error-projection orientation (Figure 4b and Figure 5b) using HTM. The base angle of the BK3 trajectory and the 8-shaped trajectory was set to 10°, consistent with ISO recommendations. The identification procedure described in the previous section was applied to both excitation configurations to estimate the rotary-axis PIGEs, and the resulting identification accuracies were evaluated and compared.

### 3.2. Experimental Work

**Figure 6:**
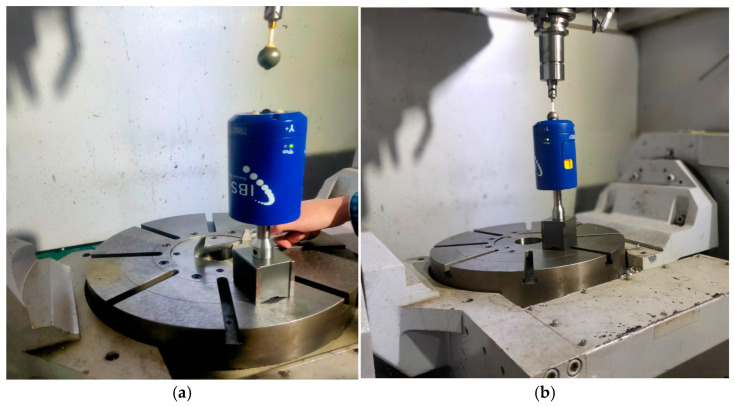
Experimental setup of the IBS precision rotary inspector device.

Experimental validation was conducted on an industrial AC-type five-axis machine tool equipped with a Siemens 840D controller, as described in Section 2.1. Before experimentation, the position-dependent geometric errors (PDGEs) of the rotary axes were compensated, and the geometric errors of the linear axes were calibrated. Consequently, the remaining geometric deviations observed during the experiments were attributed primarily to the PIGEs of the two rotary axes, which are the focus of this study. An R-test device was employed for measurement, with the master ball mounted on the spindle and the three-axis sensor probe fixed on the worktable, as shown in Figure 6. The RTCP function of the machine tool was enabled to compensate for tool length, ensuring that the master ball remained within the sensing range of the probe throughout the test.

The rotary axes were driven using NC programs generated via forward kinematic transformation of the nominal test trajectories. During measurement, the machine motion was paused at each predefined point using an M00 command, and data were recorded at 5 s intervals under quasi-static conditions. To investigate the effect of excitation projection on identification accuracy, the same physical tool motion was analyzed under different measurement-frame configurations by applying coordinate transformations to the measured data. The identification method described in the previous section was then applied to estimate the rotary-axis PIGEs for each excitation configuration. The identified PIGEs were substituted into the kinematic model to predict the tool-tip error, thereby verifying the effectiveness of the proposed identification method. Furthermore, the NC programs were modified using the computed PIGEs based on the adopted compensation strategy, and the identification accuracy was evaluated by comparing the trajectory deviations before and after compensation.

## 4. Results and Discussion

### 4.1. Simulated Results

Based on the developed kinematic model, simulation studies were conducted to evaluate the proposed identification method. The identified PIGEs are expressed in μrad for orientational errors and μm for offset errors. The results indicate that two differential iterations (first and second differentials) are sufficient to identify all rotary-axis PIGEs with satisfactory accuracy. The orientational errors $\beta_{ya}$, $\beta_{za},\;\gamma_{xc},\;and\;\gamma_{yc}$ a are accurately identified regardless of the excitation configuration or the number of differential iterations. In contrast, the offset errors $\delta_{ya},\;\delta_{za},\;\delta_{xc},\;and\;\delta_{yc}$ exhibit strong dependence on the excitation projection and are accurately identified only after the second differential under specific excitation configurations. In Table 3 and Table 4, respectively for both the BK3 and 8-shaped trajectories, the complete and accurate identification of all eight PIGEs cannot be achieved under the X direction excitation projection. However, when the excitation projection is oriented along the Y direction, all PIGEs are accurately identified after the second differentials. Multiple simulation cases with different preset PIGE values consistently exhibit the same trend, confirming that identification accuracy is governed by the excitation projection rather than by the trajectory itself. This behavior can be explained by the kinematic structure of the AC-type machine tool. The A and C axes rotate about the X and Z directions, respectively; therefore, an excitation projection along the Y direction avoids alignment with the rotary-axis screw directions and leads to improved observability of the offset-related PIGEs.

**Figure 7:**
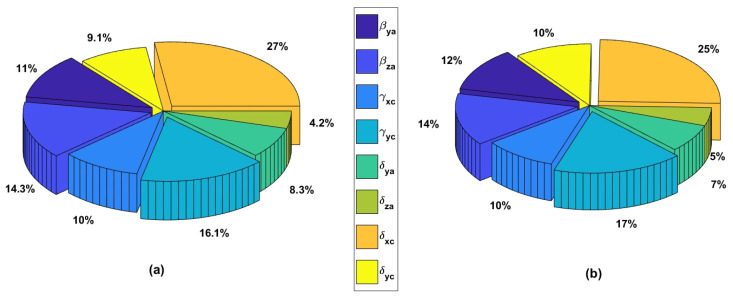
Global sensitivity percentages based on the BK3 trajectory: (**a**) In X direction; and (**b**) Y direction.

To further investigate the sensitivity characteristics of the proposed identification model, a global sensitivity analysis based on the Sobol method was performed following the procedure described by Li et al. [48]. Using the BK3 trajectory, the sensitivity results indicate that $\delta_{xc},\;\gamma_{xc},\;\beta_{za},\;and\;\beta_{ya}$ (in ascending order) have the greatest influence on the model output, while $\delta_{ya}$ exhibits the lowest sensitivity, as shown in Figure 7. The sensitivity distributions remain nearly identical for different excitation projections, demonstrating that the model structure is consistent and that the observed identification differences arise primarily from excitation conditioning rather than parameter sensitivity imbalance.

The Sobol sensitivity analysis shows that no single PIGE dominates the model output and that sensitivity distributions remain nearly identical under different excitation projections. Therefore, the identification differences in Table 3 and Table 4 are not caused by sensitivity imbalance but by excitation conditioning. Unfavorable excitation projections reduce parameter observability through geometric alignment with the measurement frame, confirming that identification accuracy is governed by excitation projection relative to machine kinematics rather than by trajectory shape.

**Figure 8:**
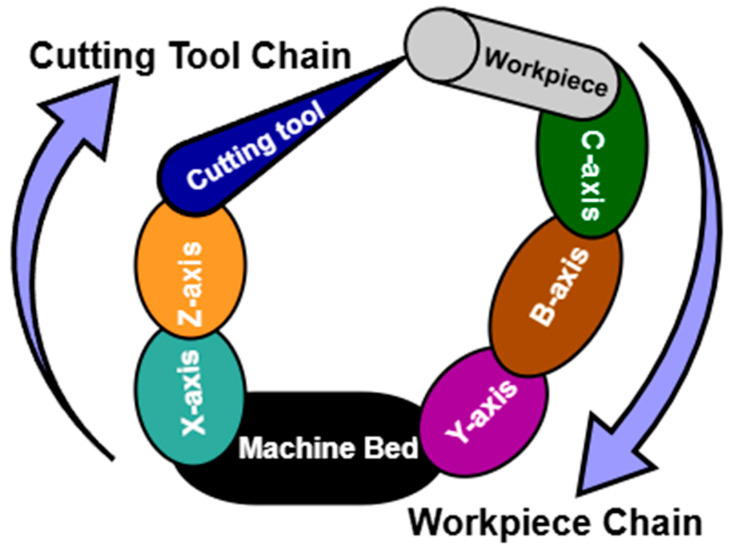
The topology of the BC-type machine tool.

To further validate the relationship between excitation projection and machine-tool configuration, additional simulations were conducted on a BC-type five-axis machine tool [49], whose topology is shown in Figure 8. In this configuration, the B and C axes rotate about the Y and Z directions, respectively, leaving the X direction free of rotary motion. Consequently, excitation projections along both the X and Y directions were analyzed through coordinate transformation.

Using parameter definitions consistent with the AC-type model, where orientational and offset errors are denoted as $S_{ij}$, respectively, the results presented in Table 5 and Table 6 show a reversed trend compared to the AC-type machine. Specifically, accurate identification of all PIGEs is achieved after the second differential when the excitation projection is aligned with the X direction, whereas identification accuracy deteriorates for the Y direction excitation projection. These results confirm that the identifiability of rotary-axis PIGEs is strongly dependent on the relative relationship between excitation projection orientation and the machine-tool kinematic configuration.

### 4.2. Experimental Results

The proposed identification method was experimentally implemented following the procedure described in the Section 3 of this work. Experimental results were obtained for both the BK3 and 8-shaped trajectories under different excitation projection configurations. As shown in Table 7, the identified orientational (squareness) errors $\beta_{ya}$, $\beta_{za},\;\gamma_{xc},\;and\;\gamma_{yc}$ obtained from the BK3 trajectory exhibit high consistency between the two excitation projections, with discrepancies of less than 1 μm. Moreover, the results from the first and second differential iterations remain stable for these parameters. In contrast, the identified offset errors show noticeable differences between excitation projections, indicating that their identifiability is strongly influenced by excitation conditioning.

Similar behavior is observed for the 8-shaped trajectory, as presented in Table 8. The orientational errors remain consistent across excitation projections, while the offset errors differ significantly, except for those identified after the second differential under the Y direction excitation projection. Notably, the offset errors obtained from the second differential under this excitation configuration are highly consistent for both trajectories, with differences below 1 μm. These results indicate that the second differential, combined with a favorable excitation projection, yields a reliable estimation of the actual rotary-axis PIGEs. Overall, the experimental results are in good agreement with the simulation findings. Minor discrepancies observed in the identified PIGE values, particularly for the second differential results, may be attributed to residual thermal effects, trajectory-following errors, and other unavoidable experimental disturbances.

**Figure 9:**
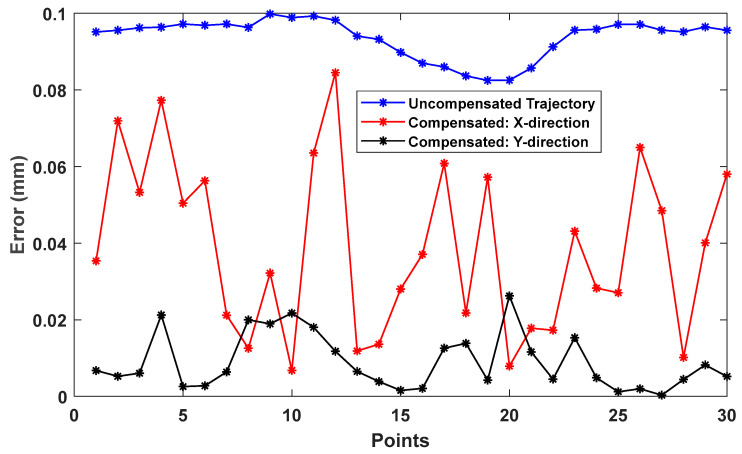
Compensation results of the BK3 trajectory.

**Figure 10:**
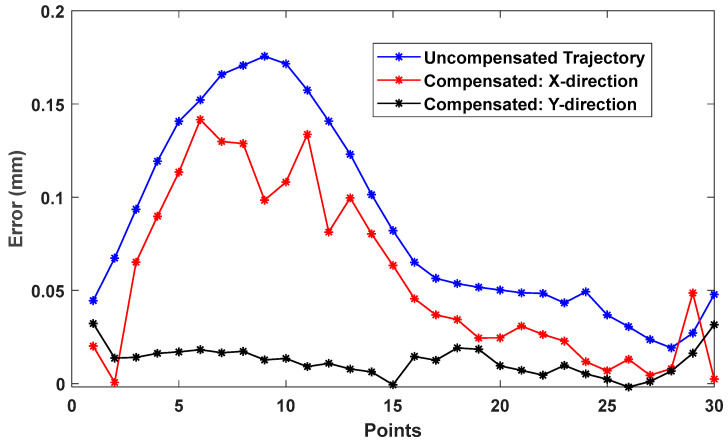
Compensation results of the 8-shaped trajectory.

To further evaluate the effectiveness of the experimentally identified PIGEs, the NC programs were modified using the proposed compensation strategy. The second differential results from each excitation projection were applied, and the trajectory deviations before and after compensation were evaluated using the RMSE. For the BK3 trajectory, the RMSE between the compensated and uncompensated trajectories is 0.0483 mm for the X direction excitation projection and 0.0932 mm for the Y direction excitation projection (shown in Figure 9). Similarly, for the 8-shaped trajectory, the RMSE values are 0.0591 mm and 0.1086 mm for the X and Y direction excitation projections, respectively, as shown in Figure 10. The larger RMSE reductions obtained using the PIGEs identified under the Y direction excitation projection indicate superior compensation performance.

A higher RMSE reduction reflects improved compensation effectiveness; therefore, the experimental results confirm that the PIGEs identified from the second differential under the Y direction excitation projection provide the most accurate representation of the actual rotary-axis geometric errors. These findings further validate that identification accuracy is governed by the relationship between excitation projection and machine-tool configuration rather than by the physical test trajectory itself. In addition, the relatively large squareness errors observed in the C axis for both trajectories may be attributed to mechanical wear.

The general applicability of the proposed method is examined through simulation and experimental studies. As shown in the results, simulations on AC- and BC-type five-axis machine tools demonstrate that identification accuracy depends on excitation projection relative to the kinematic configuration rather than on the physical placement of the rotary axes. Experimental validation on an AC-type tilting-table machine tool further confirms the practical applicability of the method, with compensation results supporting the effectiveness of the identified rotary-axis PIGEs.

Table 9 compares the proposed method with existing approaches. Unlike conventional methods that require multiple setups or test trajectories, the proposed trajectory-based excitation identifies all eight rotary-axis PIGEs in a single setup using a double differential strategy. This reduces experimental complexity and measurement time while maintaining high accuracy, as confirmed by experimental compensation results, highlighting the method’s efficiency, robustness, and industrial applicability.

In summary, the proposed identification method was successfully validated through experimental implementation. Although the same physical test trajectories were executed, the identification accuracy varied significantly with excitation projection. Both simulation and experimental results demonstrate that accurate identification of rotary-axis PIGEs is achieved after the second differential when the excitation projection is favorably aligned with the machine-tool kinematic configuration. Compensation results based on RMSE evaluation further confirm the effectiveness of the proposed method.

## 5. Conclusions

The identification of rotary-axis position-independent geometric errors (PIGEs) is essential for improving the accuracy of five-axis machine tools. Although several studies have addressed this problem, identification accuracy remains strongly dependent on the measurement strategy and computational scheme. In this study, a trajectory-based identification method using a single setup is proposed, and the influence of excitation projection on identification accuracy is systematically investigated. An error model was established, and the proposed double differential identification method was implemented and validated through both simulation and experimental studies. The results demonstrate that two differential iterations are sufficient to reliably identify the rotary-axis PIGEs. While minor discrepancies between simulated and experimental results were observed, primarily due to thermal effects, trajectory-following errors, and other dynamic disturbances, the experimental findings consistently indicate that the PIGEs obtained from the second differential under a favorable excitation projection reflect the actual geometric errors of the rotary axes.

For the experimentally investigated AC-type machine tool, the PIGEs identified under the Y direction excitation projection exhibited strong consistency across different test trajectories. Compensation results further confirmed their effectiveness, showing a significant reduction in trajectory errors for both the BK3 and 8-shaped trajectories when the NC programs were modified using these PIGEs. Simulation studies on both AC- and BC-type machine tools further revealed that identification accuracy is governed by the relative relationship between excitation projection and machine-tool kinematic configuration rather than by the physical test trajectory itself. These findings highlight that excitation projection plays a critical role in the observability and identifiability of rotary-axis PIGEs. Consequently, test-path selection and analysis should be performed in accordance with the machine-tool configuration to enhance identification accuracy. In addition, the proposed method requires only a single setup, which simplifies the measurement process and reduces experimental complexity compared to existing approaches. The results of this study provide practical guidelines for improving rotary-axis geometric error identification and offer new insight into excitation-conditioning mechanisms in five-axis machine tool calibration.

## Figures and Tables

**Table 1:** Measuring device specifications.

Parameters	Specifications
Probe	
Resolution	0.2 μm
Probe measurement uncertainty	U1 < 1.0 μm (within 1 mm range)
Measuring range of the Probe	3.50 mm
Sampling rate	2 kHz
Probe mounting shaft	Ø = 16 mm
Masterball	
Length of Masterball	75 mm
Masterball diameter	22 mm

**Table 2:** Parameter definitions of PIGEs.

Used Parameters	ISO 230-7 [41]	Definition
$\gamma_{yc}$	AOC	Squareness error of C axis to *Y* axis
$\delta_{yc}$	YOC	The linear offset of the C axis in the Y direction
$\delta_{xc}$	XOC	The linear offset of the C axis in the X direction
$\delta_{za}$	ZOA	The linear offset of the A axis in the Z direction
$\beta_{ya}$	COA	Squareness error of A axis to *Y* axis
$\gamma_{xc}$	BOC	Squareness error of C axis to *X* axis
$\delta_{ya}$	YOA	The linear offset of the A axis in the Y direction
$\beta_{za}$	BOA	Squareness error of A axis to *Z* axis

**Table 3:** Simulation results based on the BK3 trajectory.

	Error Parameter	$\beta_{ya}$ (μrad)	$\beta_{za}$ (μrad)	$\gamma_{xc}$ (μrad)	$\gamma_{yc}$ (μrad)	$\delta_{ya}$ (μm)	$\delta_{za}$ (μm)	$\delta_{xc}$ (μm)	$\delta_{yc}$ (μm)
	Set PIGEs	5.400	−16.200	−2.700	14.500	0. 8690	0.6983	0.9190	0.5844
Tilt in the X direction	1st Differential	5.500	−16.200	−2.700	14.500	0.6187	0.5961	0.8344	0.8485
	2nd Differential	5.500	−16.200	−2.700	14.500	1.0484	0.7640	0.8608	0.4243
Tilt in the Y direction	1st Differential	5.500	−16.200	−2.700	14.500	0.6340	0.6019	0.8233	0.8466
	2nd Differential	5.500	−16.200	−2.700	14.500	0.8680	0.6983	0.9190	0.5844

**Table 4:** Simulation results based on the 8-shaped trajectory.

	Error Parameter	$\beta_{ya}$ (μrad)	$\beta_{za}$ (μrad)	$\gamma_{xc}$ (μrad)	$\gamma_{yc}$ (μrad)	$\delta_{ya}$ (μm)	$\delta_{za}$ (μm)	$\delta_{xc}$ (μm)	$\delta_{yc}$ (μm)
	Set PIGEs	5.400	−11.400	6.300	9.000	−0.9363	0.4121	−0.2155	0.4863
Tilt in the X direction	1st Differential	5.400	−11.500	6.300	9.000	−0.0862	0.3911	−0.2622	0.0217
	2nd Differential	5.400	−11.500	6.300	9.000	−0.0908	0.4121	−0.2241	0.0756
Tilt in the Y direction	1st Differential	5.400	−11.500	6.300	9.000	−0.0382	0.3700	−0.4015	0.3588
	2nd Differential	5.400	−11.500	6.300	9.000	−0.9363	0.4121	−0.2155	0.4863

**Table 5:** Simulation results based on BK3 trajectory using BC-type (machine tool).

	Error Parameter	$S_{xb}$ (μrad)	$S_{zb}$ (μrad)	$S_{xc}$ (μrad)	$S_{yc}$ (μrad)	$\delta_{xb}$ (μm)	$\delta_{zb}$ (μm)	$\delta_{xc}$ (μm)	$\delta_{yc}$ (μm)
	Set PIGEs	−11.100	−8.200	−8.400	0.010.5	−0.7279	−0.7089	0.8213	−0.1372
Tilt in the X direction	1st Differential	−11.100	−8.200	−8.400	0.010.5	−2.7546	0.0000	2.9519	−0.1358
	2nd Differential	−11.100	−8.200	−8.400	0.0105	−0.7241	−0.7070	0.8218	−0.1368
Tilt in the Y direction	1st Differential	−11.100	−8.200	−8.400	0.010.4	−0.0979	−0.9253	0.2121	−0.2134
	2nd Differential	− 11.100	−8.200	−8.400	0.010.5	−2.7789	0.0000	2.9952	−0.1899

**Table 6:** Simulation results based on the 8-shape trajectory using BC-type (machine tool).

	Error Parameter	$S_{xb}$ (μrad)	$S_{zb}$ (μrad)	$S_{xc}$ (μrad)	$S_{yc}$ (μrad)	$\delta_{xb}$ (μm)	$\delta_{zb}$ (μm)	$\delta_{xc}$ (μm)	$\delta_{yc}$ (μm)
	Set PIGEs	8.800	−8.500	16.000	−5.600	0.3982	0.0119	−0.5524	0.1705
Tilt in the X direction	1st Differential	8.800	−8.500	16.100	−5.600	−0.0117	−0.0570	−0.3872	0.3380
	2nd Differential	8.800	−8.500	16.100	−5.600	0.3982	0.0120	−0.5524	0.1705
Tilt in the Y direction	1st Differential	8.800	−8.600	16.100	−5.600	−0.1582	−0.0928	−0.3127	0.0043
	2nd Differential	8.800	−8.600	16.100	−5.600	−0.0419	0.0118	−0.3256	−0.0378

**Table 7:** Experimental results based on the BK3 trajectory.

	Error Parameter	$\beta_{ya}$ (μrad)	$\beta_{za}$ (μrad)	$\gamma_{xc}$ (μrad)	$\gamma_{yc}$ (μrad)	$\delta_{ya}$ (μm)	$\delta_{za}$ (μm)	$\delta_{xc}$ (μm)	$\delta_{yc}$ (μm)
Tilt in the X direction	1st Differential	−2.412	−0.715	−0.493	2.3396	−9.496	−5.020	28.648	−15.254
	2nd Differential	−2.412	−0.715	−0.493	2.3396	27.774	5.602	28.427	−53.933
Tilt in the Y direction	1st Differential	−2.627	−0.696	−0.460	2.554	−4.746	−0.5221	19.0338	−14.734
	2nd Differential	−2.627	−0.696	−0.460	2.554	31.183	3.772	30.839	−59.064

**Table 8:** Experimental results based on the 8-shaped trajectory.

	Error Parameter	$\beta_{ya}$ (μrad)	$\beta_{za}$ (μrad)	$\gamma_{xc}$ (μrad)	$\gamma_{yc}$ (μrad)	$\delta_{ya}$ (μm)	$\delta_{za}$ (μm)	$\delta_{xc}$ (μm)	$\delta_{yc}$
Tilt in the X direction	1st Differential	−1.949	−0.536	−0.525	3.126	−7.7657	−8.323	20.568	25.765
	2nd Differential	−1.949	−0.536	−0.525	3.126	27.774	5.602	28.427	−53.933
Tilt in the Y direction	1st Differential	−2.142	−0.564	−0.653	3.227	15.3712	3.3143	9.331	−26.347
	2nd Differential	−2.142	−0.564	−0.653	3.227	33.140	4.040	31.620	−51.017

**Table 9:** Method comparison PIGEs identification.

Method	Number of Setups/Tests	Computational Strategy	Identified PIGEs	References
Axis line-height-based	8 circular tests	Multiple iterations	4	[26]
Differential motion	Spline trajectory	Differential (multiple iterations)	8	[37]
Adaptive identification	2 circular tests	Several mathematical expressions	8	[39]
Setup-based method	3 setups	4 mathematical expressions	8	[27]
Synchronous measurement	2 calibration spheres	Differential (single iteration)	4	[7]
Trajectory-based excitation	Single Setup	Differential (2 iterations)	8	Present studies

## Data Availability

The raw data supporting the conclusions of this article will be made available by the authors on request.
